# Identification of Potential Diagnostic and Prognostic Biomarkers for Colorectal Cancer Based on GEO and TCGA Databases

**DOI:** 10.3389/fgene.2020.602922

**Published:** 2021-01-14

**Authors:** Zhenjiang Wang, Mingyi Guo, Xinbo Ai, Jianbin Cheng, Zaiwei Huang, Xiaobin Li, Yuping Chen

**Affiliations:** ^1^Department of Gastroenterology, Zhuhai People's Hospital (Zhuhai Hospital Affiliated With Jinan University), Zhuhai, China; ^2^Zhuhai Precision Medical Center, Zhuhai People's Hospital (Zhuhai Hospital Affiliated With Jinan University), Zhuhai, China

**Keywords:** colorectal cancer, diagnostic biomarker, prognostic biomarker, GEO, TCGA

## Abstract

Colorectal cancer (CRC) is one of the most common neoplastic diseases worldwide. With a high recurrence rate among all cancers, treatment of CRC only improved a little over the last two decades. The mortality and morbidity rates can be significantly lessened by earlier diagnosis and prompt treatment. Available biomarkers are not sensitive enough for the diagnosis of CRC, whereas the standard diagnostic method, endoscopy, is an invasive test and expensive. Hence, seeking the diagnostic and prognostic biomarkers of CRC is urgent and challenging. With that order, we screened the overlapped differentially expressed genes (DEGs) of GEO (GSE110223, GSE110224, GSE113513) and TCGA datasets. Subsequent protein–protein interaction network analysis recognized the hub genes among these DEGs. Further functional analyses including Gene Ontology and KEGG pathway analysis and gene set enrichment analysis were processed to investigate the role of these genes and potential underlying mechanisms in CRC. Kaplan–Meier analysis and Cox hazard ratio analysis were carried out to clarify the diagnostic and prognostic role of these genes. In conclusion, our present study demonstrated that CCNA2, MAD2L1, DLGAP5, AURKA, and RRM2 are all potential diagnostic biomarkers for CRC and may also be potential treatment targets for clinical implication in the future.

## Introduction

Global Cancer Statistics 2018 indicates that colorectal cancer (CRC) accounts for ~10% of all diagnosed cancers and cancer-related deaths in the world each year (Bray et al., 2018). According to the data from China Cancer Registry Annual Report, the incidence and mortality of CRCs have been increasing in the past 10 years (Zheng et al., 2019). With the improvement of surgical methods and the launch of early tumor diagnosis and treatment, the current levels of diagnosis and treatment of CRC have been greatly improved. However, the prognosis of clinical CRC is still not optimistic. Many researches have shown that the occurrence and development of CRCs may be related to genetic, lifestyle, obesity, and environmental factors, while the exact etiology and the mechanism are still unclear (Bray et al., 2018). To further clarify the pathogenesis of CRCs and to improve the precision of treatment of CRCs, genetic research, study of tumor signaling pathways, and biological target therapy are continuing to deepen, which are gradually being applied in clinic. Meanwhile, molecular stratification therapy and application of biomarkers to guide prognosis and treatment decisions are also increasing (Bogaert and Prenen, 2014).

As we all know, the occurrence, development, overall survival time, and recurrence and non-recurrence of tumors are not only related to the pathological type and clinical stage of the tumor but also closely related to the expression and pathway of tumor genes (Bogaert and Prenen, 2014). More and more studies suggested that there are many abnormally expressed genes in the gene expression of CRCs, relative to normal tissues, which are closely related to the proliferation, differentiation, apoptosis, metastasis, recurrence, and survival time of CRC (Lu et al., 2012; Liu et al., 2013; Gan et al., 2018; Branchi et al., 2019). The analysis of abnormally expressed genes has very important clinical significance for the targeted therapy, prognosis analysis, and recurrence risk prediction of CRC. Currently, there have been a lot of clinical researches on tumor recurrence genes and signaling pathways, and the gene recurrent model (GRM) has been established to make up for the traditional tumor classification and staging recurrence prediction, providing more genetic information and more accurate prediction data (Chen et al., 2019; Yang et al., 2020). For example, Sun et al. (2019) found that exosomal CPNE3 showed potential implications in CRC diagnosis and prognosis. Carcinoembryonic antigen (CEA) was a recommended prognostic marker in CRC for tumor diagnosis and monitoring response to therapy (Campos-da-Paz et al., 2018). Ahluwalia et al. (2019) identified a novel 4-gene prognostic signature that had clinical utility in colorectal cancer. However, there are few clinical studies about biomarkers and gene pathways which have no risk of recurrence and tumor survival time.

In this study, we obtained advanced colorectal cancer gene profiles (GSE110223, GSE110224, and GSE113513) from the Gene Expression Omnibus. Differentially expressed genes (DEG) were identified by comparing CRC tissues with non-cancerous gastric tissues using the GEO2R online analysis. Subsequently, the DEG were analyzed according to Gene Ontology (GO), KEGG pathway enrichment analysis, coexpression, and protein–protein interaction (PPI) analyses. We then performed the overall survival analysis for candidate genes. Finally, GEPIA and UALCAN online tools were performed to associate candidate genes with colorectal cancer overall survival (OS), disease-free survival (RFS), and pathological staging analysis through the Cancer Genome Atlas [TCGA]) dataset and found that CCNA2, MAD2L1, DLGAP5, AURKA, and RRM2 could play an important role in colorectal cancer overall survival and disease-free survival, and these may be potential treatment targets for clinical implication in the future.

## Materials and Methods

### Microarray Data

**Figure 1:**
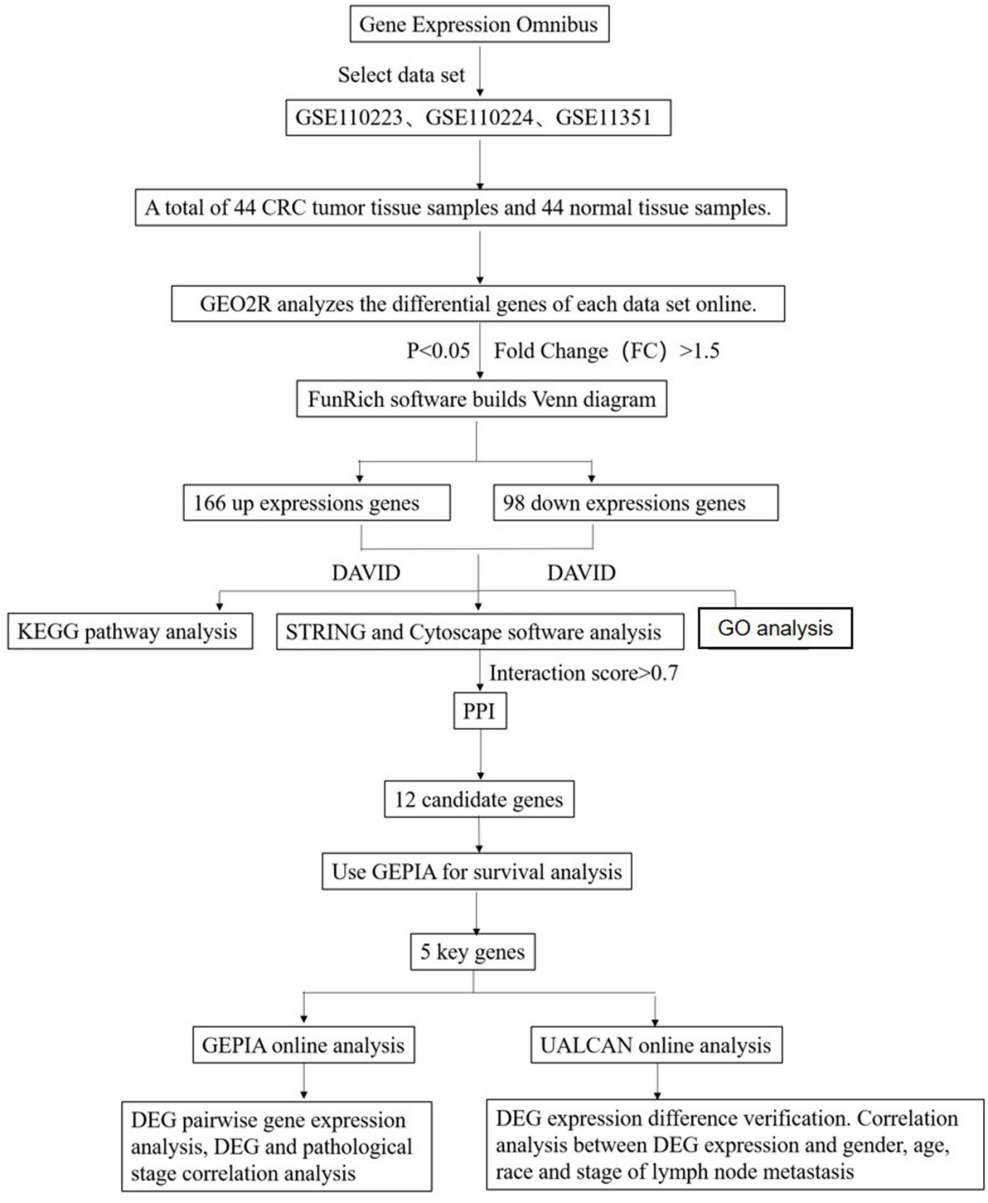
Flowchart diagram for bioinformatics analysis of publicly available datasets from both GEO and TCGA databases.

Studies from the GEO database were considered eligible satisfying the following criteria: (1) studies with CRC tissue samples, (2) studies containing information on technology and platform utilized for studies, and (3) studies including adjacent normal tissues as the control. Based on the above criteria, three datasets [GSE110223 (Vlachavas et al., 2019), GSE110224 (Vlachavas et al., 2019), GSE113513 (2018, unpublished)] are all downloaded from the GEO (Campos-da-Paz et al., 2018) database. The platform used by GSE110223 is [HG-U133A] Affymetrix Human Genome U133A Array, which includes 13 CRC tumor tissue samples and 13 normal tissue samples. The platform used by GSE110224 is [HG-U133_Plus_2] Affymetrix Human Genome U133 Plus 2.0 Array, which includes 17 CRC tumor tissue samples and 17 normal tissue samples. The platform used by GSE113513 is [PrimeView] Affymetrix Human Gene Expression Array, which includes 14 CRC tumor tissue samples and 14 normal tissue samples. A total of 44 CRC tumor tissue samples and 44 normal tissue samples were included in this study (Figure 1).

### Identification of DEGs

Using the GEOquery and limma R software packages in the Bioconductor project, the GEO2R web tool was used for identifying differential genes in selected datasets. We analyzed three GEO datasets online through GEO2R and selected the genes with *P* < 0.05 and fold change (FC) > 1.5 in the dataset as differentially expressed genes (DEGs). Then, we used FunRich_3.1.3 (Pathan et al., 2017) to make a Venn diagram and extract the differentially expressed genes common to the three datasets.

### GO and KEGG Pathway Analysis

In this study, the GO term (www.geneontology.org) and Kyoto Encyclopedia of Genes and Genomes (KEGG, www.genome.jp) approach were identified and analyzed by using DAVID v6.8 (https://david-d.ncifcrf.gov/summary.jsp) (Huang da et al., 2009a,b). The identifier and species were selected as “official_gene_symbol” and “Homo Sapiens,” respectively. The enrichment of *P* < 0.05 was set as the critical standard for significant enrichment and by using the ggplot2 package (version 3.3.1) (Wickham, 2009) and the R language (version 3.6.3, http://www.r-project.org/) to visualize the analysis results of the DAVID.

### Protein–Protein Interaction Network Analysis

STRING (version 11.0), covering 24,584,628 proteins from 5,090 organisms, and integrating known and predicted interactions between more than 932,000,000 proteins from multiple organisms including Homo sapiens (Szklarczyk et al., 2019), was used to conduct PPI network analysis on DEGs. The “Multiple proteins” button was selected, and the species were selected as “Homo Sapiens.” When the *P*-value was <0.05, the network interaction relationship is considered to be statistically significant, with interaction score >0.7 considered to be a high-confidence interaction relationship.

The Cytoscape software (version 3.6.0) (Shannon et al., 2003) was used to visualize the PPI network, and the plugin CytoHubba (Chin et al., 2014) was applied to identify the central node genes in the PPI network, and then the central node gene as a candidate DEG for the following analysis.

### The Expressions and Survival Analysis of Candidate DEGs

GEPIA (http://gepia.cancer-pku.cn/) is a database that uses standard processing methods to analyze the RNA sequencing expression data of 9,736 tumors and 8,587 normal samples from the TCGA and GTEx projects (Tang et al., 2017). GEPIA provides various functions such as tumor/normal differential expression analysis, analysis according to cancer type or pathological stage, survival analysis, correlation analysis, etc. By using the GEPIA, we performed Kaplan–Meier survival analysis on the relative expression of candidate DEGs in CRC patients and the overall survival time and disease-free survival time, with hazard ratio (HR) and corresponding 95% confidence interval. DEG related to the overall survival time and disease-free survival time was used as the study purpose DEG and conduct data analysis. Multiple-gene comparison and principal component analysis for the biomarker candidates were also conducted using the GEPIA.

UALCAN (http://ualcan.path.uab.edu/analysis.html) is an online database that uses TCGA transcriptome and clinical patient data to enable researchers to analyze the differential expression of tumor tissue and normal tissue, tumor stage, lymph node metastasis, and other related clinical parameters (Chandrashekar et al., 2017). We validated DEGs by using the UALCAN database, reanalyzed their expression differences in CRC tissue samples and normal tissue samples, and performed correlation analysis between DEG and gender, age, race, and stage of lymph node metastasis.

## Results

### Identification of DEGs

**Figure 2:**
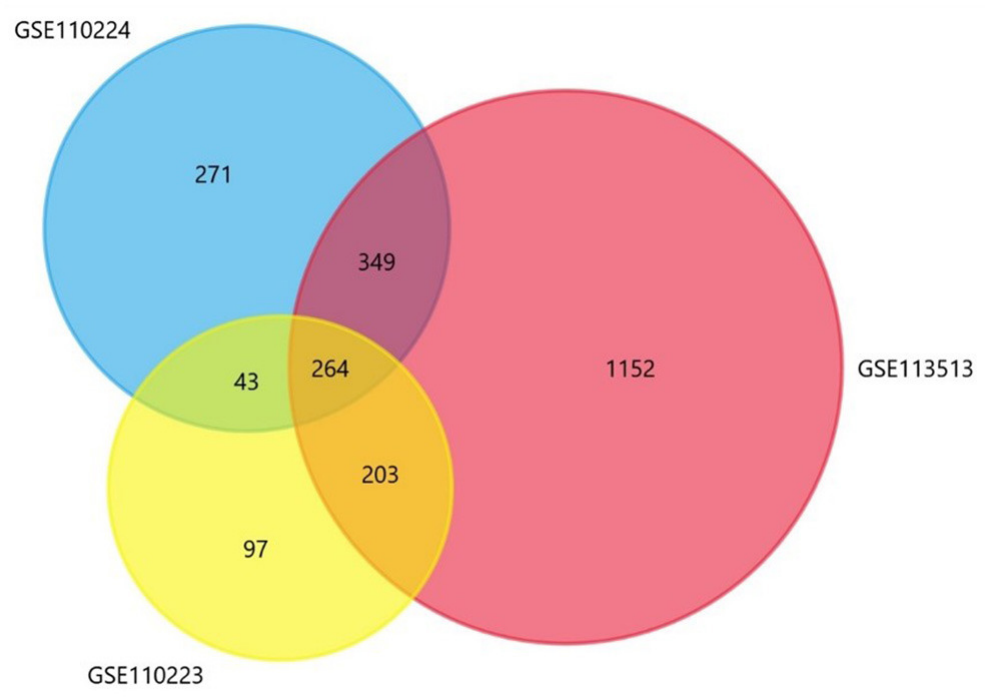
The Venn diagram shows a total of 264 co-expressed differential genes.

This study included three gene sets (GSE110223, GSE110224, GSE113513), of which GSE110223 included 13 tumor samples and 13 normal samples; GSE110224 included 17 tumor samples and 17 normal samples; and GSE113513 included 14 tumor samples and 14 normal samples. In all included datasets, compared with normal samples, there are 264 significantly different genes in all datasets (Figure 2), including 166 up-expression genes and 98 down-expression genes.

### Enrichment Analysis of DEGs

**Figure 3:**
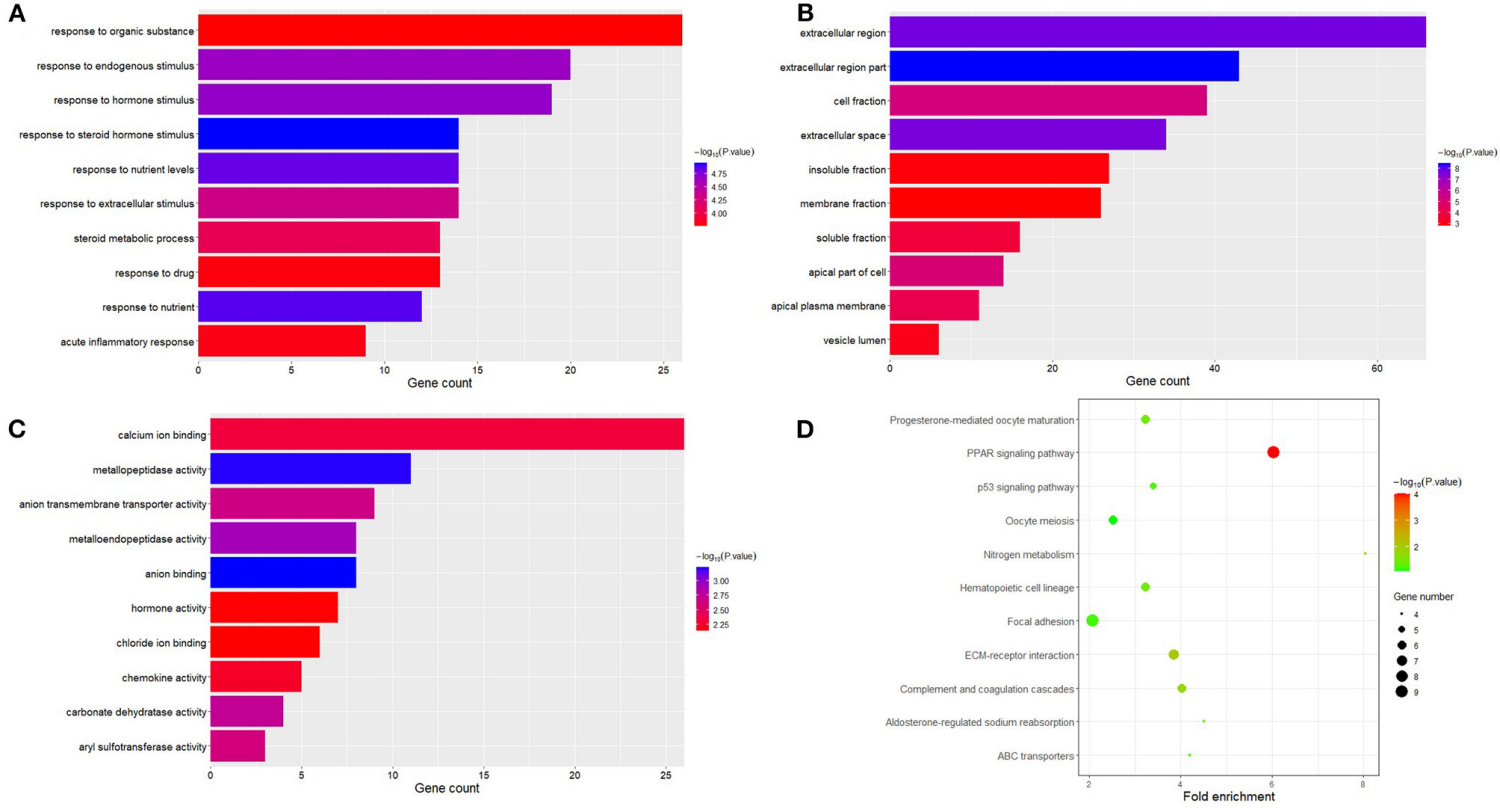
Functional analysis of differential expression genes. Biological process (BP), cell composition (CC), and molecular function (MF) are the components of GO enrichment analysis results, and each part displays 10 GO terms **(A–C)**. The result shown by KEGG is a pathway of enrichment of DGEs **(D)**.

The obtained DEGs were analyzed for functional enrichment by using DAVID. GO enrichment analysis mainly predicts the function of target genes through three aspects: biological process (BP), cell composition (CC), and molecular function (MF). By using DAVID, we found that DEG is mainly enriched in BP, including response to steroid hormone stimulus, response to nutrient, response to nutrient levels, response to hormone stimulus, response to endogenous stimulus, response to extracellular stimulus, steroid metabolic process, acute inflammatory response, response to drug, response to organic substance, etc. (Figure 3A). CC analysis includes extracellular region part, extracellular region, extracellular space, cell fraction, apical part of the cell, apical plasma membrane, soluble fraction, vesicle lumen, insoluble fraction, and membrane fraction (Figure 3B). MF analysis includes anion binding, metallopeptidase activity, metalloendopeptidase activity, carbonate dehydratase activity, anion transmembrane transporter activity, aryl sulfotransferase activity, calcium ion binding, chemokine activity, hormone activity, and chloride ion binding (Figure 3C).

At the same time, the analysis of the KEGG pathway shows that 264 DEGs are mainly enriched in eight pathways, namely, PPAR signaling pathway, ECM–receptor interaction, nitrogen metabolism, complement and coagulation cascades, hematopoietic cell lineage, progesterone-mediated oocyte maturation, aldosterone-regulated sodium reabsorption, p53 signaling pathway, focal adhesion, ABC transporters, and oocyte meiosis (Figure 3D).

### PPI Network to Identify Central Genes

**Figure 4:**
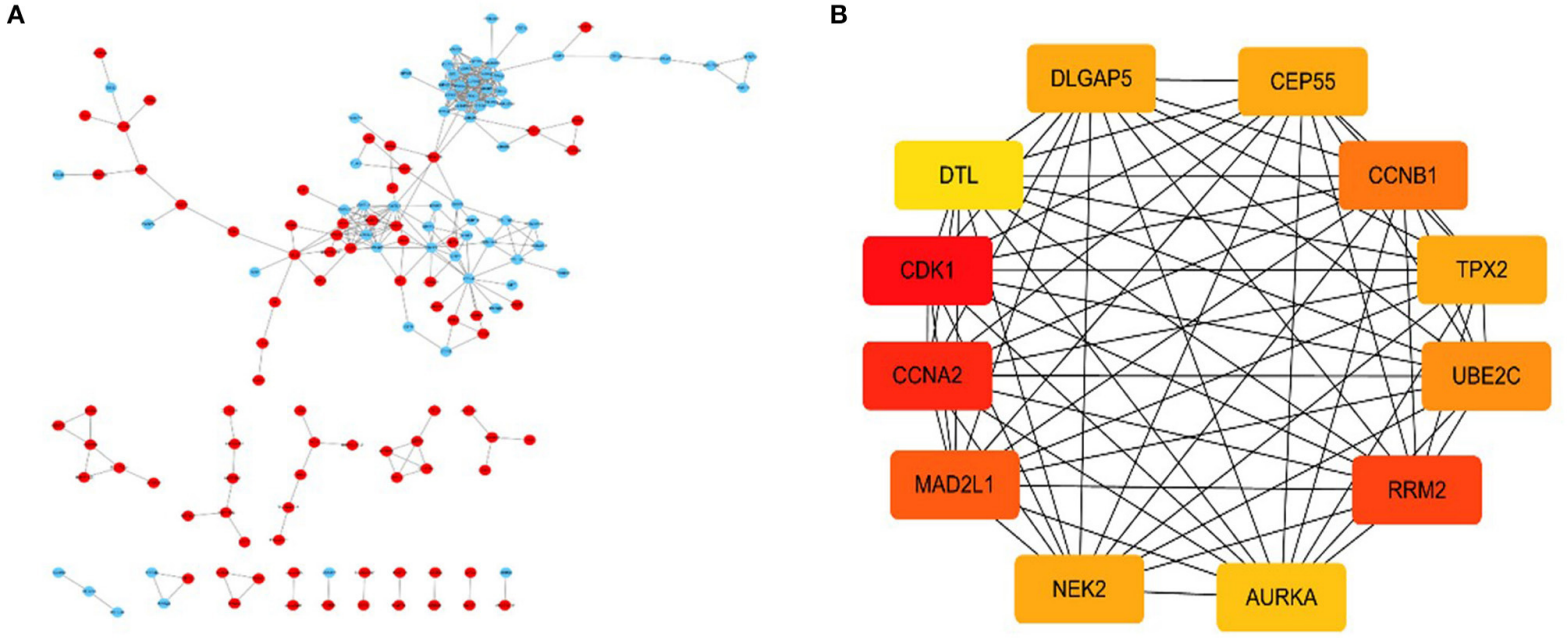
A PPI network composed of 264 DGEs, in which red is an up-expression gene, and blue is a down-expression gene **(A)**. For the first 12 central genes calculated by Cytoscape software, the red represents the degree of connectivity. The deeper the red, the higher the degree of connectivity **(B)**.

**Figure 5:**
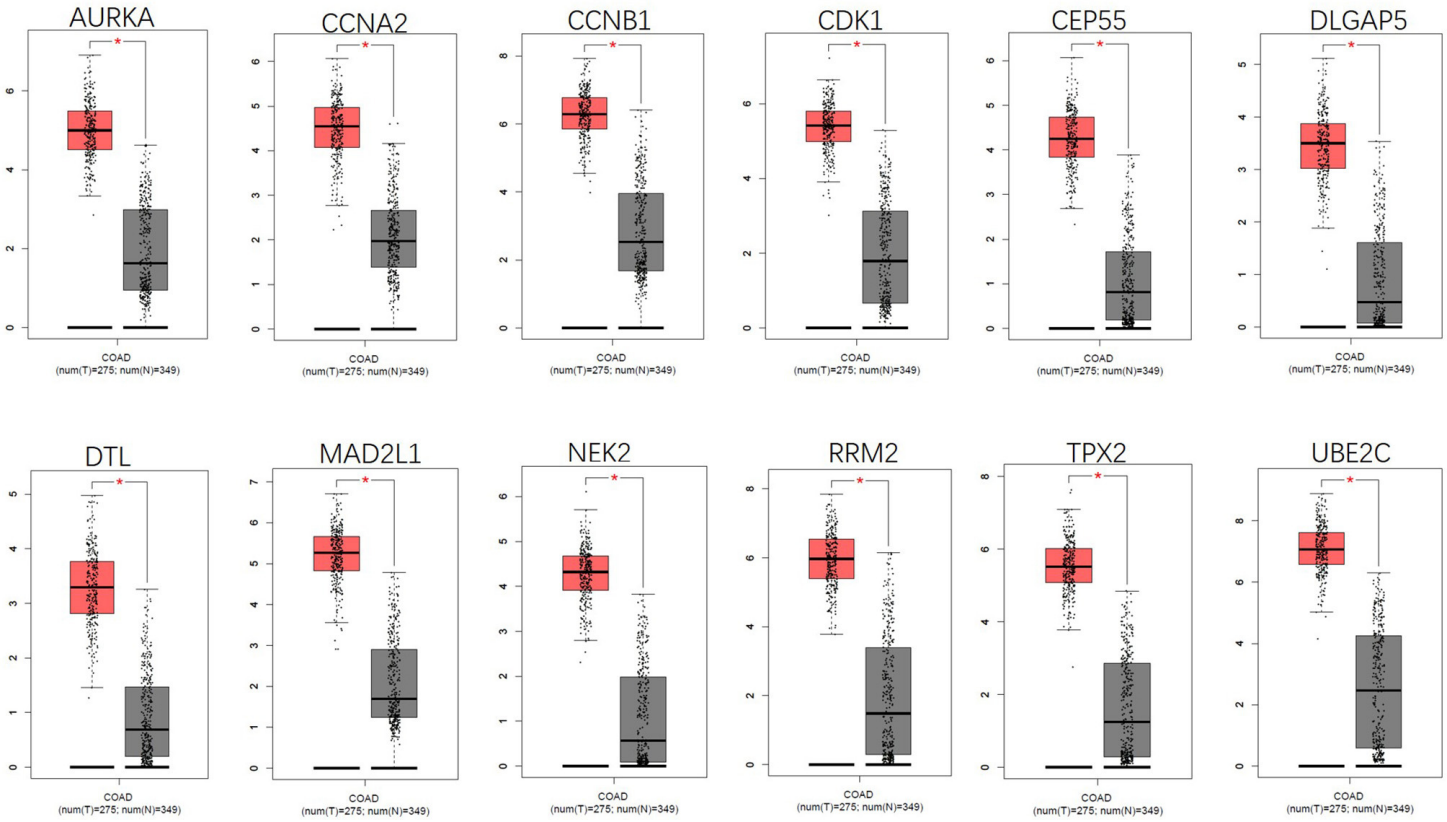
Gene expression of 12 central genes (CDK1, CCNA2, RRM2, MAD2L1, CCNB1, UBE2C, CEP55, DLGAP5, NEK2, TPX2, AURKA, and DTL) based on GEPIA.

By using the STRING database and Cytoscape 3.6.0 software, a PPI network was constructed for the 264 DGEs obtained and the central genes were determined. The PPI network constructed by STRING (Figure 4A) has a total of 262 nodes and 802 edges, and an interaction score >0.7 is considered a high-confidence interaction relationship. Using Cytoscape 3.6.0 software, we identified the top 12 genes with the most connectedness (Figure 4B). The most connected gene is CDK1, followed by CCNA2, RRM2, MAD2L1, CCNB1, UBE2C, CEP55, DLGAP5, NEK2, TPX2, AURKA, and DTL. These 12 genes can form a module. Using GEPIA, gene expression profiles of the 12 central genes between CRC tumor samples and normal samples were displayed in Figure 5.

### Overall Survival Analysis and Disease-Free Survival Analysis

**Figure 6:**
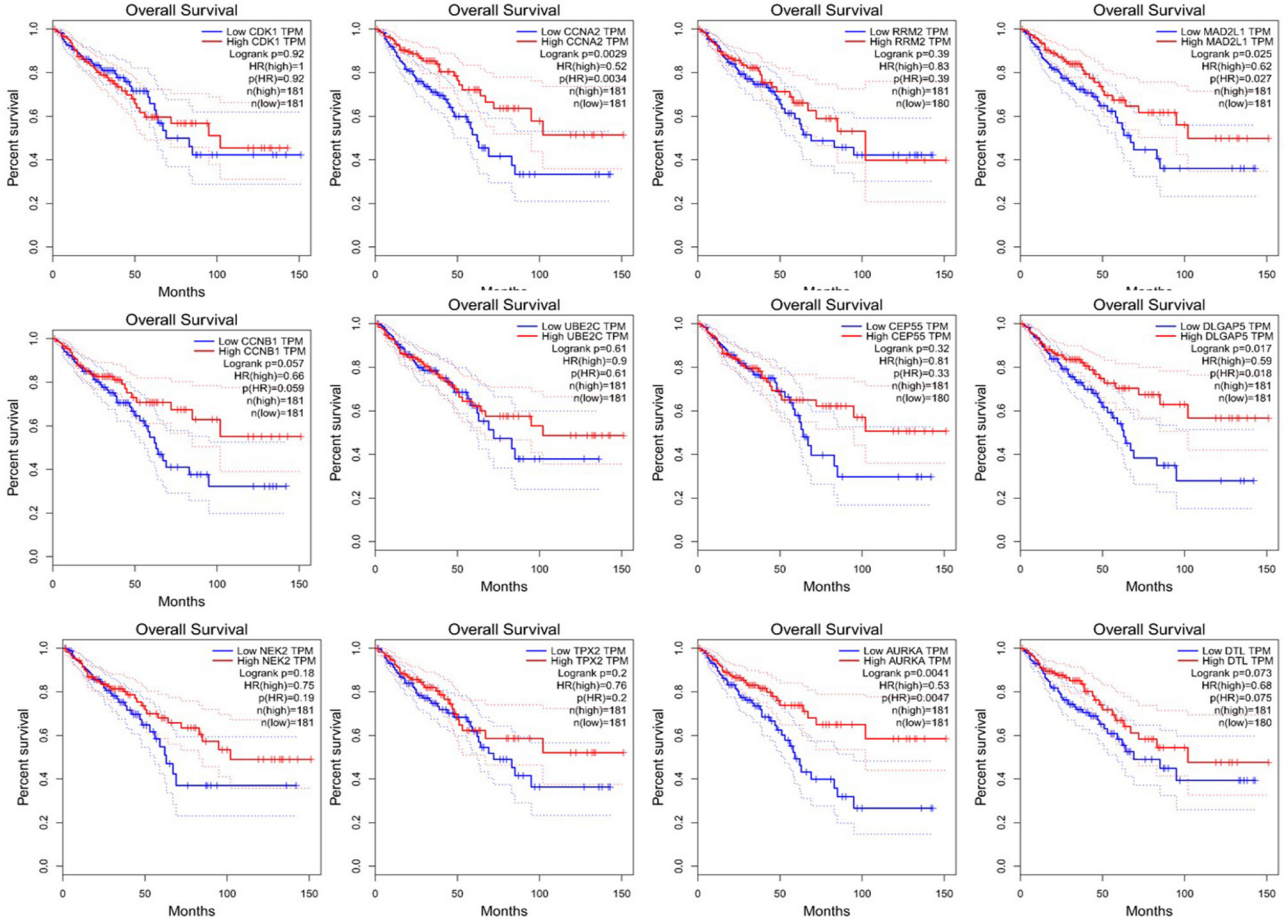
Overall survival analysis of 12 central genes (CDK1, CCNA2, RRM2, MAD2L1, CCNB1, UBE2C, CEP55, DLGAP5, NEK2, TPX2, AURKA, and DTL) based on GEPIA.

**Figure 7:**
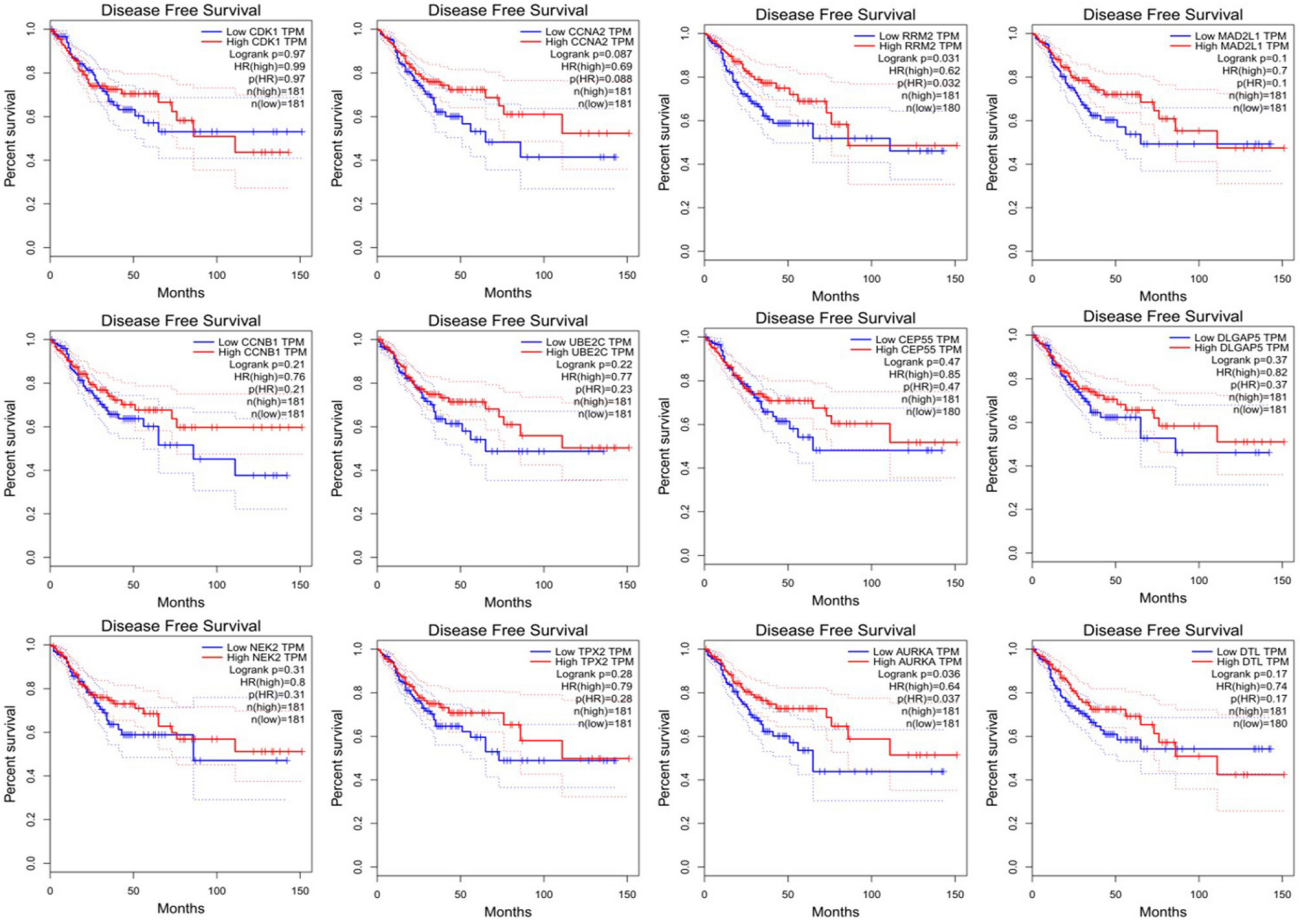
Disease-free survival analysis 12 central genes (CDK1, CCNA2, RRM2, MAD2L1, CCNB1, UBE2C, DLGAP55, DLGAP5, NEK2, TPX2, AURKA, and DTL).

Since CRC is mainly adenocarcinoma, which can account for 90%, we used the GEPIA database to analyze the overall survival and disease-free survival of colorectal adenocarcinoma on 12 central genes. We found that among the 12 central genes, CCNA2, MAD2L1, DLGAP5, and AURKA were associated with the overall survival of colorectal adenocarcinoma (*P* < 0.05) (Figure 6), and RRM2 and AURKA were associated with disease-free survival of colorectal adenocarcinoma (*P* < 0.05) (Figure 7). Therefore, this study will focus on the five genes CCNA2, MAD2L1, DLGAP5, AURKA, and RRM2.

### Correlation Analysis Based on the GEPIA

**Figure 8:**
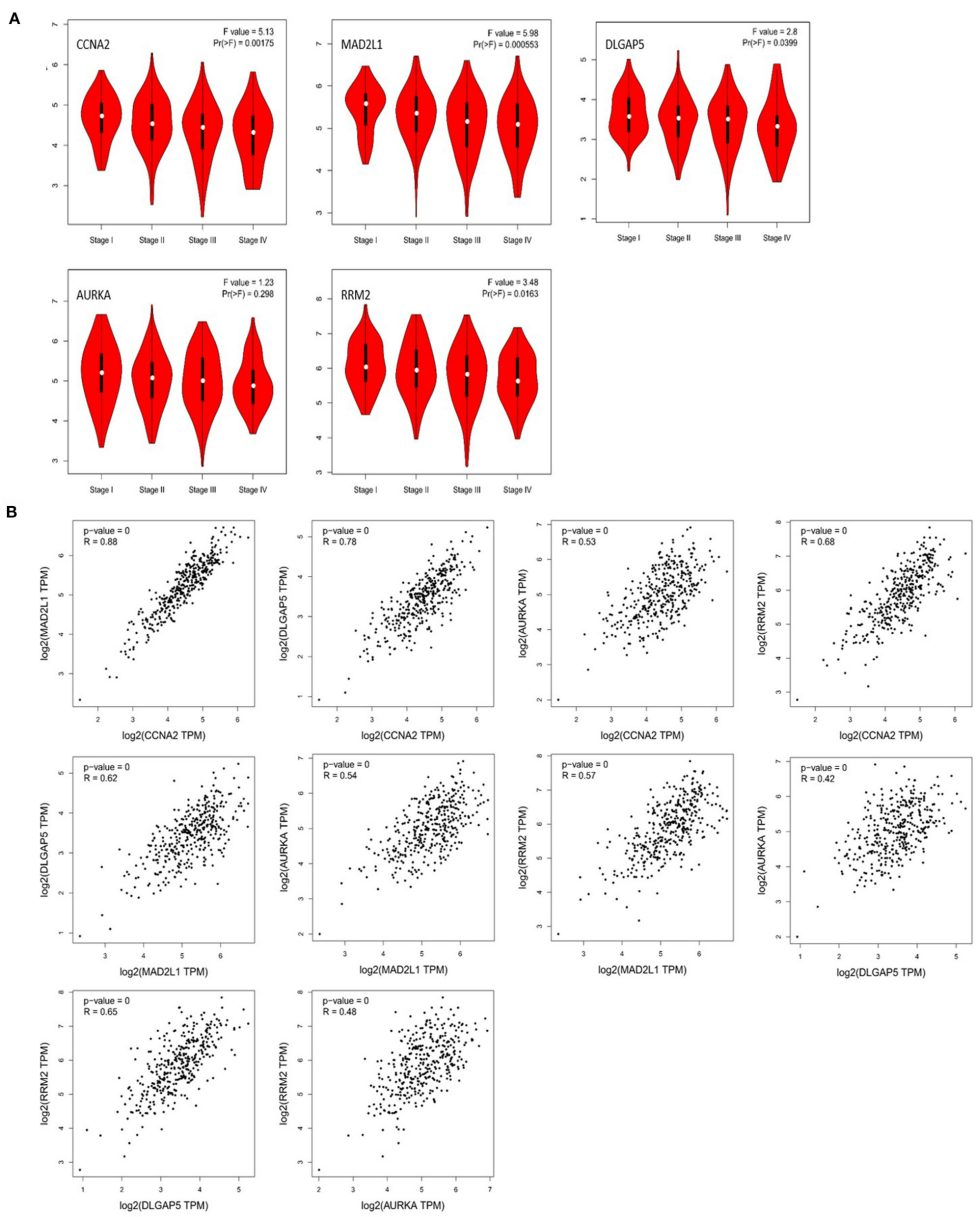
Correlation analysis between CCNA2, MAD2L1, DLGAP5, and RRM2 and the pathological stage of colorectal adenocarcinoma **(A)**. Correlation analysis of the expression of CCNA2, MAD2L1, DLGAP5, and RRM2 in colorectal adenocarcinoma **(B)**.

Through the analysis of the correlation between these five genes and the pathological staging of colorectal adenocarcinoma based on GEPIA, we found that CCNA2, MAD2L1, DLGAP5, and RRM2 are all significantly related to the pathological stage of COAD and READ (*P* < 0.05), while AURKA is associated with the colorectal gland, with no significant correlation between cancer pathological staging (*P* > 0.05) (Figure 8A). The correlation analysis of the expression of these five genes in colorectal adenocarcinoma showed that CCNA2 was highly correlated with MAD2L1 (*P* < 0.001, *R* = 0.88), and it was also correlated with DLGAP5 (*P* < 0.001, *R* = 0.78), AURKA (*P* < 0.001, *R* = 0.53), and RRM2 (*P* < 0.001, *R* = 0.68). Moderate positive correlations between MAD2L1 and DLGAP5 (*P* < 0.001, *R* = 0.62), MAD2L1 and AURKA (*P* < 0.001, *R* = 0.54), and MAD2L1 and RRM2 (*P* < 0.001, *R* = 0.57) were observed. DLGAP5 and AURKA (*P* < 0.001, *R* = 0.42) had low expression correlation, and there was a moderate positive correlation between DLGAP5 and RRM2 (*P* < 0.001, *R* = 0.65). AURKA and RRM2 (*P* < 0.001, *R* = 0.48) have a low expression correlation (Figure 8B).

### Verification of the Differential Expression of CCNA2, MAD2L1, DLGAP5, AURKA, and RRM2 and the Analysis of Related Clinical Parameters

**Figure 9:**
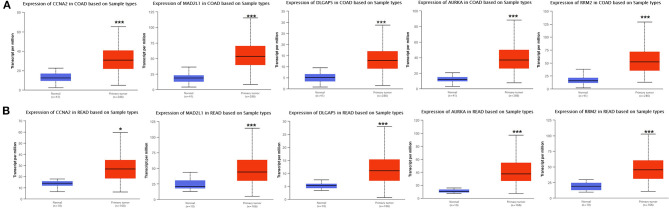
Verification of the differential expression of CCNA2, MAD2L1, DLGAP5, AURKA, and RRM2 in CRC: COAD **(A)**; READ **(B)** (*P < 0.05, ***P < 0.001).

Analysis for gene differential expression through the UALCAN software (http://ualcan.path.uab.edu/analysis.html) indicated that there were significant differences in expression of the five genes in the normal group and the tumor group, both in colon adenocarcinoma (COAD) and rectal adenocarcinoma (READ) (Figure 9).

For COAD, taking the normal samples as the reference group, the expression of these five genes all significantly increased, whether in males or in females (Figure S1A), various age groups (Figure S2A), various races (Figure S3A), and various lymph node metastasis stages (Figure S4A).

For READ, taking the normal samples as the reference group, in terms of gender (Figure S1B), the expression of these five genes was significantly increased in males or females. In terms of age (Figure S2B), CCNA2, MAD2L1, and AURKA were 21–40. The expression of DLGAP5 was not obvious in the age groups of 41–60, 61–80, and 81–100. DLGAP5 was not significantly expressed in the groups of 21–40 and 81–100. Both the 60-year-old group and the 61–80-year-old group were significantly expressed, and RRM2 was significantly expressed in all age groups. In terms of race (Figure S3B), as the sample number of Asian patients was only one case, the Asian patient sample group was not tested. By comparison, the expression of these five genes was significantly increased in both the African-American patient group and the Caucasian patient group. In terms of lymph node metastasis (Figure S4B), the expression of CCNA2 was significantly increased in N1 and N2, but not in N3. MAD2L1, DLGAP5, AURKA, and RRM2 were significantly increased in the metastasis stage of each lymph node.

### Possibility of a Five-Gene Biomarker in Diagnosis of CRC

**Figure 10:**
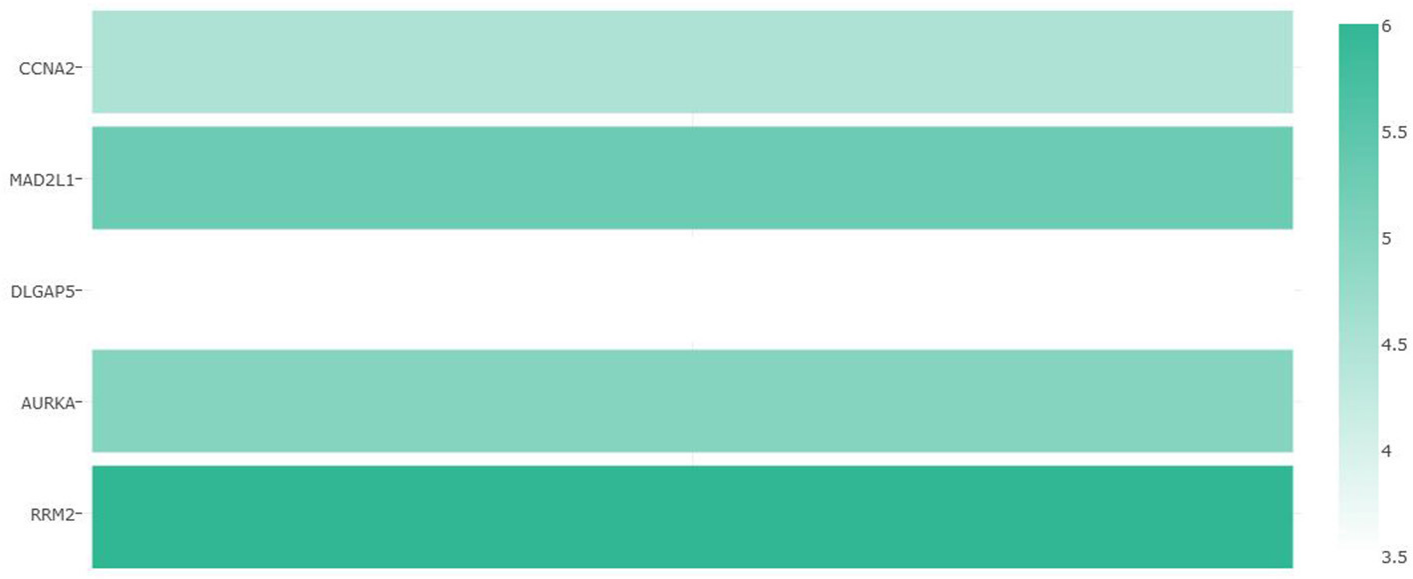
Multiple-gene comparison analysis for the five CRC biomarker candidates.

**Figure 11:**
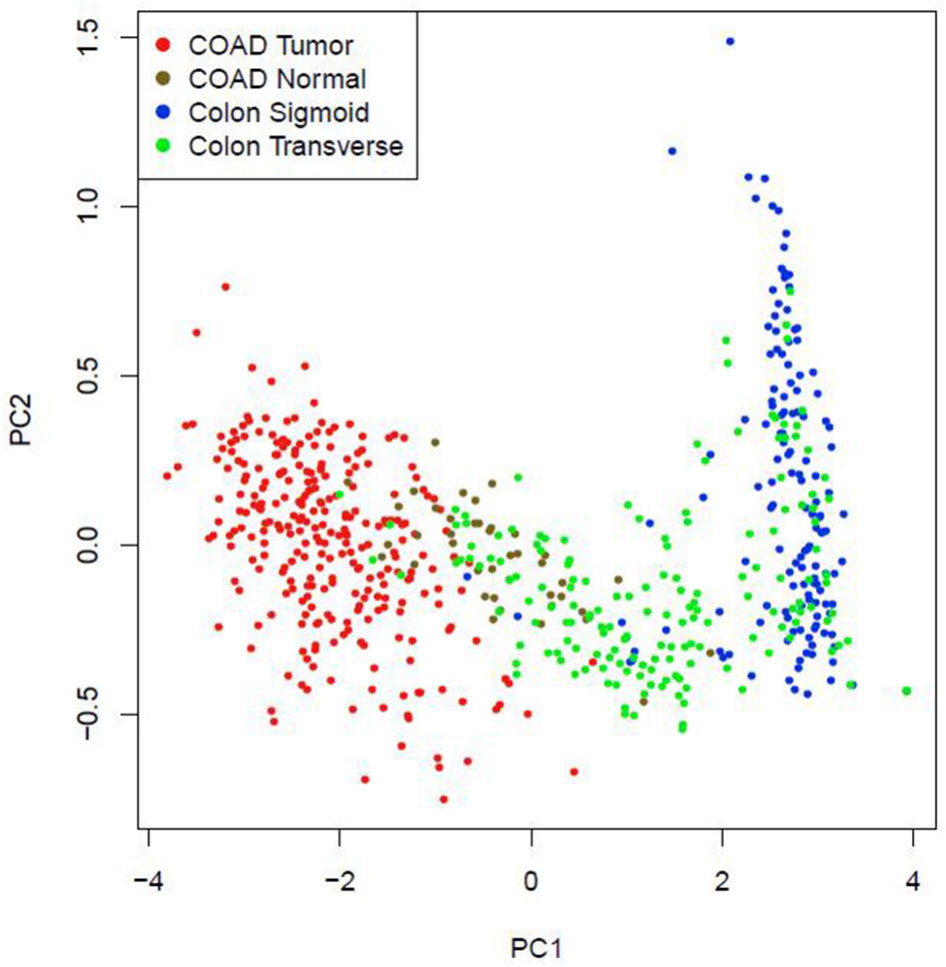
Principal component analysis of the five CRC biomarker candidates.

Multiple-gene comparison analysis for the five CRC biomarker candidates was conducted using the GEPIA, with the only tumor data (COAD). Among the five genes, RRM2 had the highest expression level, followed by MAD2L1, AURKA, CCNA2, and DLGAP5 (Figure 10). Principal component analysis of the five genes was performed with TCGA tumor data, TCGA normal data, and GTEx data (both colon-sigmoid and colon-transverse); we found that the five genes could effectively distinguish between CRC samples and normal samples (Figure 11), indicating the possibility of a five-gene biomarker in diagnosis of CRC.

## Discussion

CRC is one of the most common cancers and carries a major global health burden. Globally, among all cancers, CRC ranked third in incidence and second in mortality in 2018 (Bray et al., 2018). There is no specific clinical symptom of colorectal cancer at the early stage, but when it was found, it had become in the middle and late stages. Surgery and radiotherapy and chemotherapy at the perioperative period or adjuvant chemotherapy is still the first choice for colorectal cancers (Schmoll et al., 2012). In those with metastatic disease, the treatment repertoire has been extended to include biologically targeted agents, including monoclonal antibodies targeting EGFR, such as cetuximab or panitumumab (Tabernero et al., 2015). As a result of improved treatment options, the overall survival (OS) of patients with metastatic CRC has increased from ~1 year in the era of 5-fluorouracil (5-FU) therapy alone, to ~3 years with currently available therapies (Cremolini et al., 2015). However, recurrence and overall survival are still the challenge for the treatment of colorectal cancer in clinic. Therefore, it is crucial to identify new markers that can predict CRC recurrence, overall survival (OS), and disease-free survival (RFS), subsequently separating patients into high- or low-risk groups for enhanced efficacy in further treatment. There are many clinical studies related to tumor recurrence. In this study, we focus on the genes about overall survival (OS) and disease-free survival (DFS) in CRC. Three datasets (GSE110223, GSE110224, GSE113513) from the GEO database were introduced into the analysis; 264 DEGs were overlapped in all the three datasets including 166 upregulated DEGs and 98 downregulated DEGs. Even though the three datasets had the similar number of samples, the number of DEGs of each dataset varied greatly. GSE113513 had the largest number of DEGs, largely more than those of other two datasets.

We found that CCNA2, MAD2L1, DLGAP5, AURKA, and RRM2 were closely related to the prognosis of CRC. Among them, we found that CCNA2, MAD2L1, DLGAP5, and AURKA were related to the overall survival (OS) of colorectal tumors, while RRM2 and AURKA had the relation between disease-free survival (DFS) and colorectal cancer. CCNA2, MAD2L1, DLGAP5, and RRM2 were all significantly related to the pathological stages of colorectal cancer and were also closely related to the stage of lymph node metastasis. There was a study that revealed that the expression of CCNA2 in CRC tissues is higher than that in normal tissues. The knockdown of CCNA2 could significantly suppress CRC cell growth by impairing cell cycle progression and inducing cell apoptosis (Gan et al., 2018). Our study showed that the abnormal expression of CCNA2 was also significantly related to the overall survival time, pathological stage of the tumor, and lymph node metastasis. There were articles that showed that CCNA2 is an important sign to judge the poor prognosis of the tumor, as it also highly expressed in pancreatic cancer, breast cancer, lung cancer, and other tumors (Gao et al., 2014; Peng et al., 2018; Brcic et al., 2019). MAD2L1, DLGAP5, and AURKA are the key genes for spindle assembly. When these genes are abnormally expressed, they will cause chromosome mismatch and other genetic problems during mitosis (Ooi et al., 2012). What is worse is that the unstable gene expression will eventually lead to cancer (Wassmann and Benezra, 2001; Weaver and Cleveland, 2005). Clinical studies had shown that DLGAP5 was related to the invasion and migration of CRC (Branchi et al., 2019); besides, DLGAP5 expression was also related to overall survival and lymph node metastasis but had no correlation with disease-free survival. It is an important measure of poor prognosis. Previous studies had verified that MAD2L1, DLGAP5, and AURKA were highly expressed in CRC (Chuang et al., 2016; Branchi et al., 2019; Ding et al., 2020). In our study, we found that these abnormally expressed genes not only induce the occurrence and development of tumors but also are significantly related to the overall survival, pathological staging of tumors, and tumor lymph node metastasis. When it comes to the expression of RRM2, studies showed that it related to the depth of invasion, degree of differentiation, disease-free survival (RFS), and metastasis of CRC (Lu et al., 2012; Liu et al., 2013). Our study showed that RRM2 was associated with disease-free survival of colorectal adenocarcinoma and was an important target gene predicted after tumor treatment.

In summary, it can be seen that CCNA2, MAD2L1, DLGAP5, RRM2, and AURKA are significantly related to the overall survival prognosis, disease-free survival, pathological stage, and lymph node metastasis stage of CRC, which are also important indicators for the evaluation of the prognosis of CRC and the evaluation of further treatment. Principal component analysis of the five genes indicated that they could effectively distinguish between CRC samples and normal samples. In order to increase the accuracy of diagnosing CRC, we suggested that the five biomarker candidates as a five-gene biomarker for diagnosis of CRC.

Once we understand the site where abnormal gene expression induces tumors, more targeted drugs will be applied. Most importantly, it provides a theoretical basis for future gene-level treatment of tumors and can achieve more precise targeted therapy. It is also important to guide the development of genetic kits and the non-invasive diagnosis of colorectal tumors.

## Data Availability Statement

The datasets presented in this study can be found in online repositories. The names of the repository/repositories and accession number(s) can be found in the article/Supplementary Material.

## Ethics Statement

Ethical review and approval was not required for the study on human participants in accordance with the local legislation and institutional requirements. Written informed consent for participation was not required for this study in accordance with the national legislation and the institutional requirements. Written informed consent was not obtained from the individual(s) for the publication of any potentially identifiable images or data included in this article.

## Author Contributions

YC and XL designed and directed the research. ZW and MG conducted data collection, data statistics, and article writing for the research. JC, ZH, and XA assisted in related literature search. All authors contributed to the article and approved the submitted version.

## Conflict of Interest

The authors declare that the research was conducted in the absence of any commercial or financial relationships that could be constructed as a potential conflict of interest.
