# Supplementary material for: Identification of Potential Diagnostic and Prognostic Biomarkers for Colorectal Cancer Based on GEO and TCGA Databases
Source: Front Genet. 2021 Jan 14;11:602922. doi: 10.3389/fgene.2020.602922 (PMC7841465; doi:10.3389/fgene.2020.602922)
Supplement: Supplementary file 1 [file Data_Sheet_1.docx]

**Identification of Potential Diagnostic and Prognostic Biomarkers** **for Colorectal Cancer Based on GEO and TCGA Databases**

**Figure S1**.Figure S1. The correlation analysis between the gene expression of CCNA2, MAD2L1, DLGAP5, AURKA and RRM2 in CRC and gender: COAD (A); READ (B) (* P<0.05, ** P<0.01, *** P <0.001,- P>0.05).

**Figure S2**. The correlation analysis between the gene expression of CCNA2, MAD2L1, DLGAP5, AURKA and RRM2 in CRC and age: COAD (A); READ (B) (* P<0.05, ** P<0.01, *** P <0.001,- P>0.05).

**Figure S3**. The correlation analysis between the gene expression of CCNA2, MAD2L1, DLGAP5, AURKA and RRM2 in CRC and race: COAD (A); READ (B) (* P<0.05, ** P<0.01, *** P <0.001,- P>0.05)

**Figure S4**. The correlation analysis between the gene expression of CCNA2, MAD2L1, DLGAP5, AURKA and RRM2 in CRC and the stage of lymph node metastasis: COAD (A); READ (B) (* P<0.05, ** P<0.01, ** * P<0.001,- P>0.05).


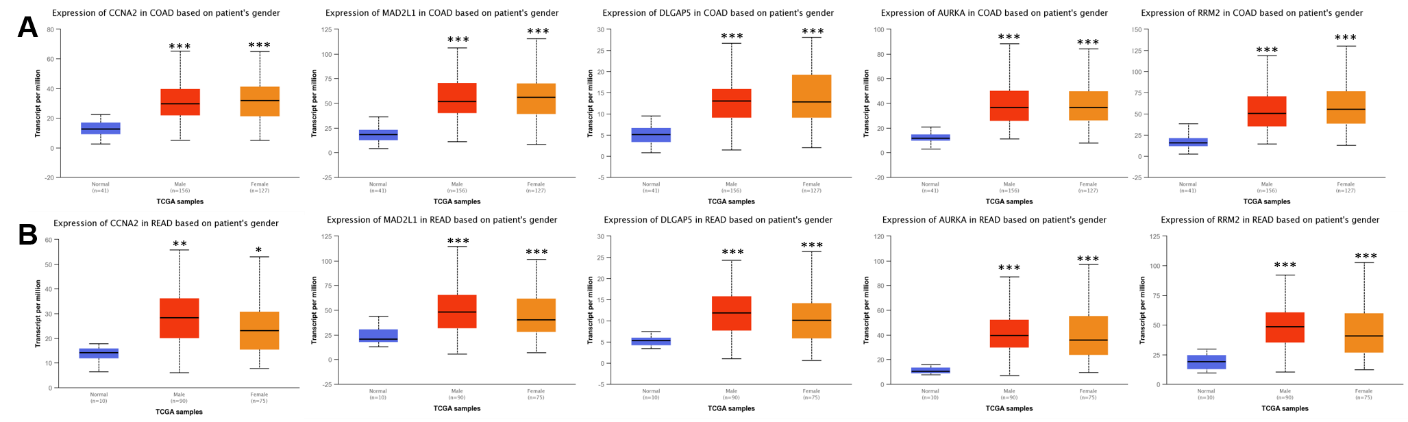


Figure S1


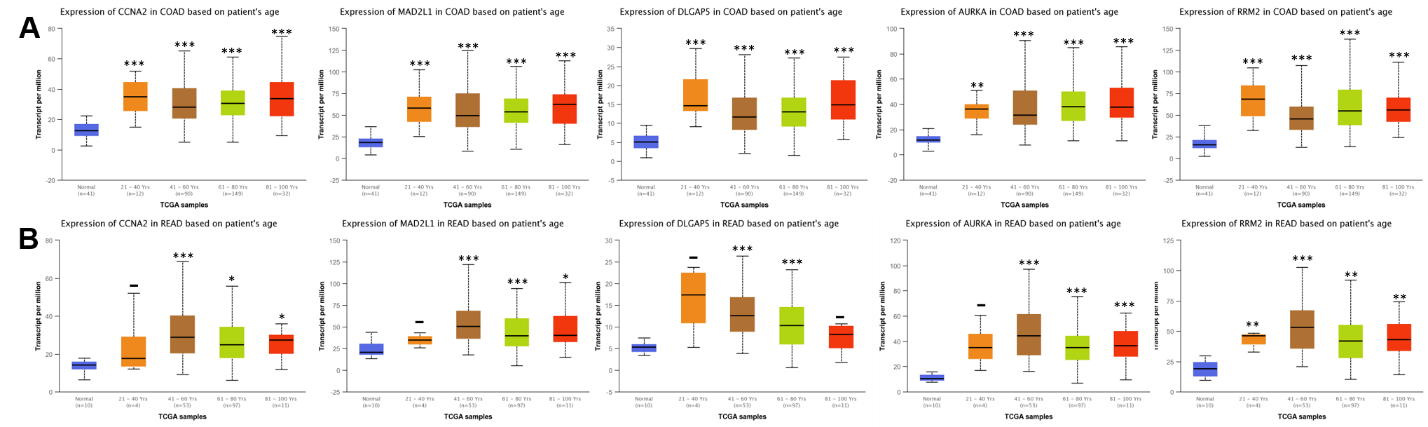


Figure S2


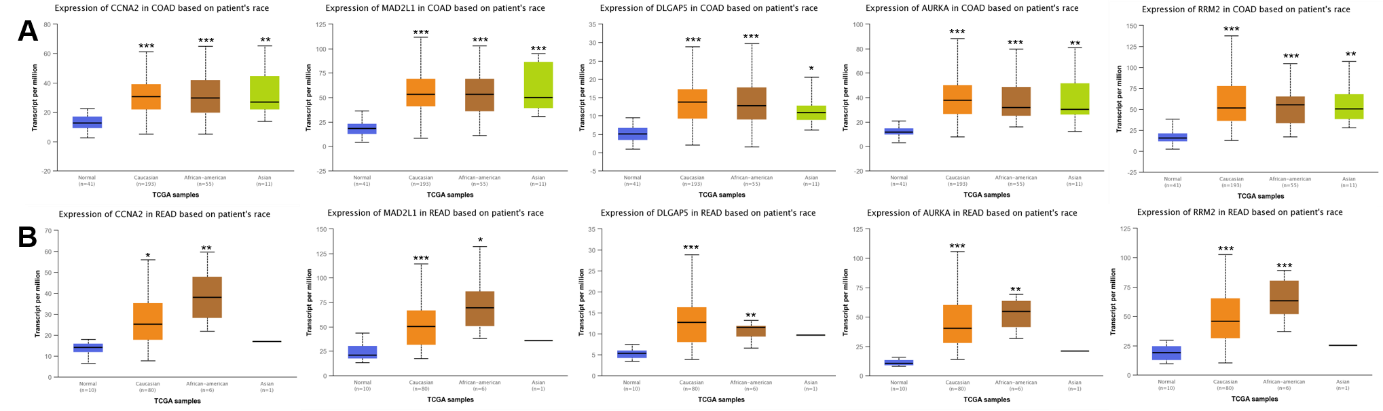


Figure S3


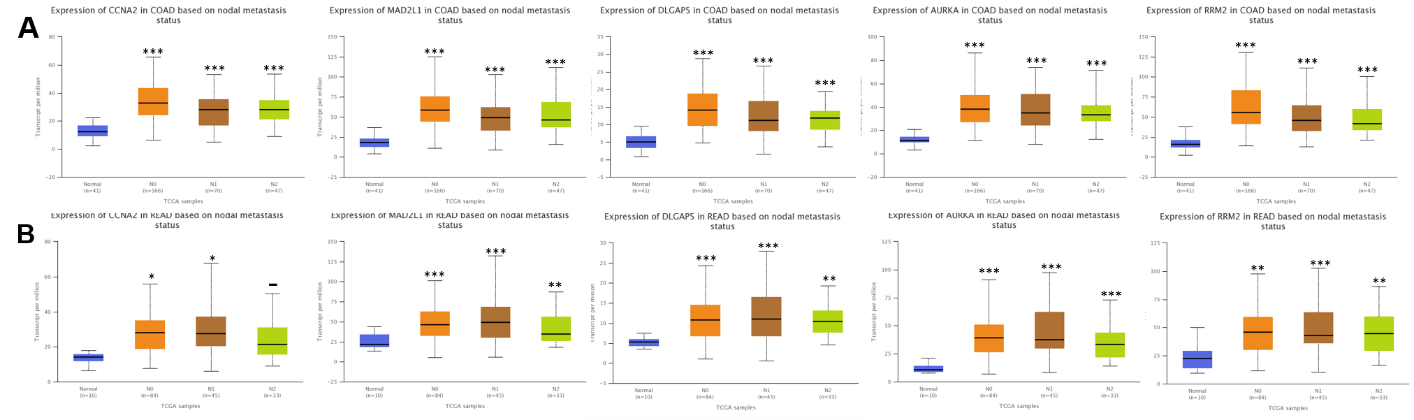


Figure S4
